# Comparative Study of the Sedative and Anti-nociceptive Effects of Sacrococcygeal Epidural Administration of Romifidine, Lidocaine, and Romifidine/Lidocaine in the Dromedary Camel

**DOI:** 10.3389/fvets.2022.891581

**Published:** 2022-06-27

**Authors:** Mohamed Marzok, Adel I. Almubarak, Sherief M. Abdel-Raheem, Sabry El-khodery, Turke Shawaf, Mahmoud Kandeel

**Affiliations:** ^1^Department of Clinical Sciences, College of Veterinary Medicine, King Faisal University, Al-Hofuf, Saudi Arabia; ^2^Department of Surgery, Faculty of Veterinary Medicine, Kafr El Sheikh University, Kafr El Sheikh, Egypt; ^3^Department of Public Health, College of Veterinary Medicine, King Faisal University, Al-Hofuf, Saudi Arabia; ^4^Department of Animal Nutrition and Clinical Nutrition, Faculty of Veterinary Medicine, Assiut University, Assiut, Egypt; ^5^Department of Internal Medicine, Infectious Diseases and Fish Diseases, Faculty of Veterinary Medicine, Mansoura University, Mansoura, Egypt; ^6^Department of Biomedical Sciences, College of Veterinary Medicine, King Faisal University, Al-Hofuf, Saudi Arabia; ^7^Department of Pharmacology, Faculty of Veterinary Medicine, Kafr El Sheikh University, Kafr El Sheikh, Egypt

**Keywords:** anti-nociception, camels, epidural, neuraxial, romifidine, lidocaine, sedation

## Abstract

In a randomized prospective study, comparative sedative and anti-nociceptive effects of epidural administration of romifidine (RO), lidocaine (LD), and a combination of romifidine-lidocaine (ROLD) in camel were evaluated. Eighteen healthy adult dromedary camels were assigned randomly to three treatment groups (*n* = 6), each receiving 50 μg/kg of RO, 0.30 mg/kg of LD, or a combination of both RO and LD. All treatments were expanded in 0.9% sterile normal saline solution to a final dose volume of 20 ml and administered directly into the sacrococcygeal space. After epidural injection of each treatment, the onset time, duration, anatomical extension of anti-nociception, and sedation were documented. Anti-nociception was tested at different areas using a pinprick test and artery forceps pinching at the perineum and inguinal area. RO and ROLD treatments resulted in mild to severe sedation and complete bilateral analgesia with loss of sensation in the tail, perineum, scrotum in males, vulva in females, the caudal aspect skin of the upper hind limb, and inguinal region (udder in females and the prepuce in males). The anatomic extent of anti-nociception reached the chest cranially and the footpad distally. Camels who received LD showed the shortest duration (*P* < 0.001) to the onset of perineal anti-nociception (3.67 ± 0.33 min) followed by those who received RO LD (4.00 ± 0.37 min) and RO (6.67 ± 0.33 min), respectively. RO and ROLD resulted in significantly (*P* < 0.001) longer periods of analgesia (158.33 ± 4.01 min and 165 ± 3.87 min, respectively) than LD (75.83 ± 3.27). An epidural RO and ROLD would appear to produce a very effective and acceptable anti-nociceptive effect in the perineal and inguinal regions of camels.

## Introduction

In veterinary practice, caudal epidural analgesia is a valuable tool as a potential substitute for general anesthesia for various surgical and obstetrical interventions in ruminants, especially under field conditions. Furthermore, caudal epidural analgesia can provide efficient analgesia with superior therapeutic efficacy in the management of pre- and postoperative pain in the hind limbs, pelvis and caudal regions in ruminants (1, 2). Therefore, the utility of this technique in the field of veterinary anesthesia has garnered increased clinical attention over the last decade (3–5). Caudal epidural administration of local analgesic agents (usually a 2% lidocaine solution) has been widely reported for surgical interventions of the tail, anus, rectum, perineum and urogenital system (vulva, vagina, urethra and bladder) in the camel (6). However, most local analgesics provide anti-nociception for a relatively short period and may need to be readministered to allow the completion of longer procedures. Furthermore, these agents block sensory, motor, and sympathetic fibers non-specifically, resulting in hind limb weakness and sometimes recumbency (7, 8). For procedures requiring long-term analgesia, extradural or epidural administration of longer-acting analgesic drugs may be more suitable. These drugs include alpha-2 adrenoceptor agonists, dissociative anesthetics, steroidal and non-steroidal compounds, and opioids that discriminately block sensory fibers, resulting in considerable analgesia with a reduced likelihood of disruption of pelvic limb motor function (9–14). These drugs are either administrated alone or in various combinations (15, 16).

The Arabian (dromedary) camel (*Camelus dromedarius*) is a very valuable species in Arabian Gulf countries; however, very little research has investigated the effectiveness of anesthetic techniques in this species, and many aspects of anesthesia are unknown. Romifidine (RO) is one of the alpha-2 adrenoceptor agonists most regularly used in equine practice and is useful as a sedative and analgesic in camels (17). In recent years, RO has been used epidurally to induce perineal analgesia for spinal (neuraxial) epidural analgesia in many species of animals such as horses and other equines, goats, cattle and buffalo (2, 18–20). To the authors' knowledge, no research has investigated the use of RO as an epidural analgesia in the dromedary camel. Therefore, the purpose of this research was to evaluate and compare the sedative and anti-nociceptive effects of RO, LD, and a combination ROLD when administered in the camel's epidural space.

## Materials and Methods

### Experimental Camels and Drugs

Eighteen adult healthy dromedary camels, nine each of the Magateer and Majaheem breeds, were selected for this study. Nine were non-pregnant females, and nine were intact males. Mean ± SD age was 5.7 ± 1.6 years, the mean weight was 391.7 ± 30.9 kg, and the mean body condition score was 3.8 ± 0.6 (21).

All experimental camels were reared at King Faisal University Camel Research Center (Al-Hofuf, KSA) and judged healthy based on clinical examination and a full biochemical and hematological assessment. The exclusion criteria were camels with unhealthy condition, body condition scores below 3, unpalpable sacrococcygeal space, previously received epidural injections or local blocks in the perineal region and those with skin diseases in the area of interest. Each animal was identified by a visual ear tag inserted into the left ear. The females were housed together in a box/pen, and the males were separated from the females and held individually in separate boxes/pens. All camels were fed grass hay supplemented with concentrate. They were allowed free access to water while feed was withdrawn for 24 h before the experiment.

RO (10 mg/ml, Boehringer Ingelheim, Vetmedica, Germany), LD (20 mg/mL, preservative-free and vasoconstrictor-free, Pharmaceutical Solutions Industry, Jeddah, KSA) and 0.9% sodium chloride (Pharmaceutical Solution Industries, Al-Khobar, KSA) were administered epidurally.

### Trial Design and Experimental Procedure

The Animal Care Committee of King Faisal University reviewed and approved the study protocol in correspondence with Saudi Arabian ethical codes for studies on experimental animals (approval no. KFUREC/ 2021-03-01). Camels were assigned randomly to three treatment groups, with six camels in each group (three non-pregnant females and three males: three Magateer and three Majaheem). All trials were conducted outdoors in a quiet environment and natural daylight, with a target temperature of ~30°C, and all camels were restrained in the Cush position. Camels were allowed to acclimate to their surroundings for 20 min in the stall before evaluation.

At the beginning of each experiment, each camel was weighed, and its body temperature, ruminal contractions, heart rate (HR), and respiratory rate (RR) were measured. The fiber over the sacrococcygeal area was clipped and the area scrubbed with povidone–iodine. Each group received one of three treatments (equal volumes) administered into the epidural space over approximately 30 s. Treatments were 50 μg/kg RO, 50 μg/kg RO co-administered with preservative-free and vasoconstrictor-free LD, 0.30 mg/kg, and 0.30 mg/kg LD. All treatments were expanded in 0.9% sterile normal saline solution to provide a total dose volume of 20 ml and administered directly into the extradural space between the last sacral and first coccygeal vertebrae (the sacrococcygeal space), using a 16-gauge, 6-cm hypodermic needle. The space was identified by moving the tail up and down while palpating the depression between the fifth sacral and first coccygeal vertebrae. The needle was inserted into the skin surface at an angle of 45–50° with the median plane. Correct needle placement was verified by lack of resistance to injection and detecting negative pressure via the hanging drop technique. All treatments were prepared by one person (MK) and administered by the same investigators (MM and AM), who were blinded to the drug used. Following drug administration, the camels were raised and directed into a chute and monitored for any drug-related side effects.

### Evaluation of Clinical Parameters After Epidural RO, ROLD and LD Administration

The onset time, duration, and anatomical locations of anti-nociception and sedation were documented after epidural injection of each drug. Clinical parameters measured included HR, RR, ruminal contractions and rectal temperature (RT). Also measured were scores for sedation, anti-nociception and ataxia. Anal sphincter relaxation, ballooning of the caudal part of the rectum, penile prolapse in males and frequency of urination were recorded immediately (time 0) pre-administration and at 5, 15, 30, 45, 60, 90, 120, 150, 180, and 210 mins and 6 and 12 h post-administration. HR was assessed by auscultation as beats per minute, RR was evaluated as the number of rib movements per minute, ruminal contraction was measured by auscultation, and RT was measured rectally with a veterinary digital thermometer.

### Assessment of Analgesia

Analgesia was tested at anatomical points, including the base of the tail, anus, vulva, perineum, caudal aspect of the thigh, inguinal region (udder in females and prepuce in males), flank, lateral abdominal wall, chest, shoulder, neck, dorsal metatarsal area, and the footpad using a pinprick test (a 22-gauge, 2.5-cm-long hypodermic needle) through the skin into the deep tissues at the above-mentioned points. For each time point, the needle was inserted bilaterally at a slightly different location. The skin prick wounds were sprayed with povidone-iodine solution. When pinpricks elicited no response, pinching using artery forceps was used to test for a high degree of analgesia. Pinching was applied only at the perineum and inguinal area. The intensity of anti-nociception was graded on a scoring system from 0 to 3 [as described previously in buffalo (2)]: 0, no analgesia (vigorous response to a painful stimulus, such as the forceful motion of the animals' limb); 1, mild analgesia (moderate response, such as turning the head toward the site of stimulation); 2, moderate analgesia (very weak and intermittent response); and 3, complete analgesia (no response to a painful stimulus). Time to onset of perineal analgesia was documented every minute after the epidural injection by evaluating the animal's response to pinpricks and artery forceps pinching. The duration of perineal analgesia (in min) was estimated as the time between the loss and reappearance of a response to the pinprick and pinching stimuli.

### Assessment of Sedation

The sedative effect was assessed according to a modified four-point descriptive scale in each camel for each treatment (17): 0 = no sedation (aware, alert, maintaining normal position of the head, ear, eyelids, neck, lips and tongue and sensitive to tapping on metal bar close to the animal's head); 1 = mild sedation (reduced alertness, slight drop of head, ear and lips, palpebral ptosis, protrusion of the tongue out the mouth with slightly decreased reaction to tapping on metal bar close to the animal's head); 2 = moderate sedation (sluggishness, an obvious drop of the head, ear and lips, more protrusion or hanging of the tongue out the mouth, neck deviation and occasional response to tapping on metal bar close to the animal's head); 3 = deep sedation (marked sluggishness, drop of head and lips, palpebral ptosis, deviation of the neck, pronounced ear tip separation and lower ear carriage and lack of response to tapping on metal bar close to the animal's head).

The time from the epidural administration to the start of sedation was considered the sedation onset time. The duration of sedation (in min) was estimated as the time from the start of sedation to the return of the sedation score to zero.

### Assessment of Motor Effects (Ataxia)

Ataxia was monitored by observing the position of the animal's hind limbs, the extent of knuckling over fetlock joints, and the animal's attempt to lie down. Ataxia was scored on a simple 4-point scale (20): 0 = normal, 1 = slight or mild (slight or intermittent wide stance of hind legs, slight swaying or stumbling, but able to walk), 2 = moderate (pronounced stumbling, frequent wide stance of hind legs, frequent fetlock knuckling, walking with extreme incoordination, attempting to lie down but easily persuaded to stand), or (3) severe (assuming a Cush position and unable to be raised). The same observer (AM) evaluated anti-nociception, ataxia and sedation in all animals and was completely unaware of the treatments administered.

### Statistical Analysis

All statistical analyses were carried out using a commercial software program (SPSS for windows, United States). The Kolmogorov–Smirnov test was used to determine whether the data were normally distributed. For variables presented as scores, (analgesia, sedation and ataxia), non-parametric Kruskal–Wallis test, with *post hoc* Dunn's multiple comparison test was used at each time point. The results were presented as median and range. However, for normally distributed continuous data, results were presented as mean ± SE. Data for onset, duration of analgesia and sedation were analyzed using a one-way ANOVA with *post hoc* Duncan multiple range test. Data of heart rate, respiratory rate, rectal temperature, and ruminal contractions were found normally distributed; therefore, general linear model with repeated measure ANOVA was used to assess the effect of within group (time), between groups (treatment), and time x treatment interaction. For this purpose, Wilks' Lambda was used. Whenever Wilks' Lambda test was found significant (*P* < 0.05), one-way ANOVA with *post hoc* Tukey's HSD test was used at each time point to assess which group was significantly different. For all analyses, result was considered significant at *P* < 0.05

## Results

The epidural injection was easily and successfully performed in all camels without any adverse side effects observed after epidural injection. No precipitation occurred in the ROLD mixture.

**Figure 1:**
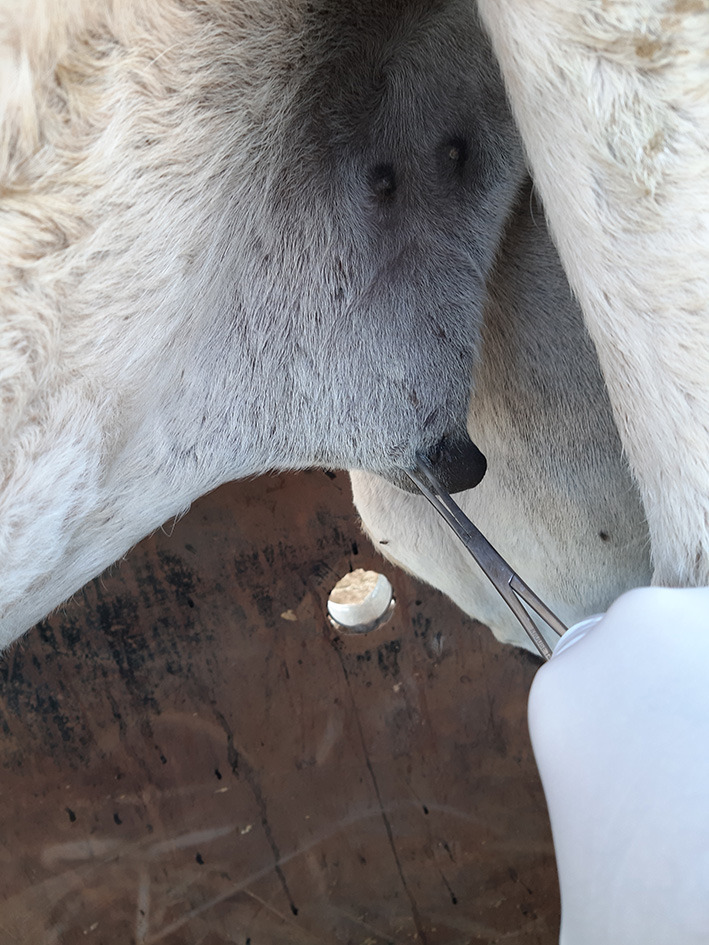
Pinching using artery forceps showing a complete analgesia of the prepuce in male camel post-epidural injection of romifidine (RO) (50 μg/kg).

All three epidural treatments induced complete bilateral analgesia with loss of sensation in the tail, perineum, scrotum in males, vulva in females, and the skin of the caudal aspect of the upper hind limb (Table 1). Furthermore, all camels that received RO and ROLD showed complete analgesia of the inguinal region (udder in females and the prepuce in males) (Figure 1), and the anatomic extent of anti-nociception reached the chest cranially and the footpad distally (Table 1). All three treatments resulted in a maximum degree of anti-nociception (score = 3), but different onset and length times and at different locations (Table 1).

**Table 1:** Anti-nociception scores (median and range) in the tail, perineum, and caudal aspect of the upper hind limbs, the dorsal metatarsal and foot pad regions, and the flank and chest regions (response to “pin-prick” stimulation), pre-epidural and post-epidural injection of romifidine (RO) (50 μg kg^−1^), romifidine-lidocaine (ROLD) (50 μg and 0.3 mg kg^−1^) and lidocaine (LD) (0.3 mg kg^−1^) in camels (*n* = 18).

**Treatment**	**Time post-administration (minutes)**												
	**0**	**5**	**15**	**30**	**45**	**60**	**90**	**120**	**150**	**180**	**210**	**360**	**720**
**The tail, perineum, and caudal aspect of the upper hind limbs**													
RO	0 (0)	0 (0–0)^a^	3 (2–3)	3 (3–3)	3 (3–3)	3 (3–3)	3 (3–3)^a^	3 (3–3)^a^	2.5 (2–3)^a^	0 (0–1)^a^	0 (0–1)^a^	0 (0–0)	0 (0–0)
ROLD	0 (0)	2 (2–2)^b^	3 (3–3)	3 (3–3)	3(3–3)	3 (3–3)	3 (3–3)^a^	3 (3–3)^a^	3(2–3)^a^	3 (2–3)^b^	2 (1–3)^b^	0 (0–0)	0 (0–0)
LD	0 (0)	2 (2–3)^b^	3 (3–3)	3 (3–3)	3 (3–3)	3 (2–3)	0 (0–1)^b^	0 (0–0)^b^	0 (0–0)^b^	0 (0–0)^a^	0 (0–0)^a^	0 (0–0)	0 (0–0)
*P* value	1	<0.01	0.37	1	1	0.12	<0.001	<0.001	<0.01	<0.01	<0.01	1	1
**The dorsal metatarsal and the foot pad regions**													
RO	0 (0)	0 (0–0)	2.5 (2–3)^a^	3 (2–3)^a^	3 (2–3)^a^	3 (2–3)^a^	3 (2–3)^a^	2.5 (2–3)^a^	2 (2–3)^a^	1 (1–2)^a^	0 (0–1)	0 (0–0)	0 (0–0)
ROLD	0 (0)	0 (0–0)	2 (1–3)^a^	3(2–3)^a^	3 (2–3)^a^	3 (2–3)^a^	3 (2–3)^a^	3 (2–3)^a^	2.5 (2–3)^a^	2 (1–2)^a^	0.5 (0–1)	0 (0–0)	0 (0–0)
LD	0 (0)	0 (0–0)	0 (0–0)^b^	0 (0–0)^b^	0 (0–0)^b^	0 (0–0)^b^	0 (0–0)^b^	0 (0–0)^b^	0 (0–0)^b^	0 (0–0)^b^	0 (0–0)	0 (0–0)	0 (0–0)
*P* value	1.00	1.00	<0.01	<0.01	<0.01	<0.01	<0.01	<0.01	<0.01	<0.01	0.12	1.00	1.00
**The flank and chest regions**													
RO	0 (0)	0 (0–0)	3 (2–3)^a^	3 (3–3)^a^	3 (3–3)^a^	3(3–3)^a^	3 (2–3)^a^	3 (2–3)^a^	2 (2–3)^a^	1 (1–2)^a^	0 (0–1)	0 (0–0)	0 (0–0)
ROLD	0 (0)	0 (0–0)	3 (2–3)^a^	3(2–3)^a^	3 (2–3)^a^	3(3–3)^a^	3 (2–3)^a^	3 (1–2)^a^	3 (2–3)^a^	2 (1–2)^a^	0 (0–1)	0 (0–0)	0 (0–0)
LD	0 (0)	0 (0–0)	0 (0–0)^b^	0 (0–0)^b^	0 (0–0)^b^	0(0–0)^b^	0 (0–0)^b^	0 (0–0)^b^	0 (0–0)^b^	0 (0–0)^b^	0 (0–0)	0 (0–0)	0 (0–0)
*P* value	1.00	1.00	<0.01	<0.01	<0.001	<0.001	<0.01	<0.01	<0.01	<0.01	0.16	1.00	1.00

a, b *Variables with different superscript letters at the same column are significantly differ at P < 0.05*.

Camels who received LD showed the shortest duration (*P* < 0.001) to the onset of perineal anti-nociception (3.67 ± 0.33 min) followed by those who received RO LD (4.00 ± 0.37 min) and RO (6.67 ± 0.33 min), respectively. The duration of perineal anti-nociception is significantly different between the RO, RO LD and LD groups (*P* < 0.001) and being longer in RO and ROLD (158.33 ± 4.01 and 165 ± 3.87 mins, respectively) in comparison with LD (75.83 ± 3.27) (Table 2).

**Table 2:** Onset and length of perineal anti-nociception, sedation, ataxia (mean ± SE), following epidural_injection of romifidine (RO) (50 μg kg^−1^), romifidine-lidocaine (ROLD) (50 μg and 0.3 mg kg^−1^) and lidocaine (LD) (0.3 mg kg^−1^) in camels.

**Variable**	**RO**	**ROLD**	**LD**	***P* value**
**Anti-nociception**				
Onset (mean ± SE)	6.67 ± 0.33^a^	4.00 ± 0.37^b^	3.67 ± 0.33^b^	<0.001
Duration (mean ± SE)	158.33 ± 4.01^a^	165 ± 3.87^a^	75.83 ± 3.27^b^	<0.001
**Sedation**				
Onset (mean ± SE)	6.33 ± 0.49^a^	6.5 ± 0.43^a^	0^b^	<0.001
Duration (mean ± SE)	160 ± 6.33^a^	141.67 ± 5.43^b^	0^c^	<0.001
**Ataxia**				
Onset (mean ± SE)	12.83 ± 0.79^b^	14 ± 0.53^b^	20.83 ± 1.53^a^	<0.001
Duration (mean ± SE)	149.5 ± 4.92^a^	144.17 ± 6.25^a^	80.09 ± 3.16^b^	<0.001

a, b *Means with different superscript letters at the same row are significantly differ at P < 0.05*.

**Figure 2:**
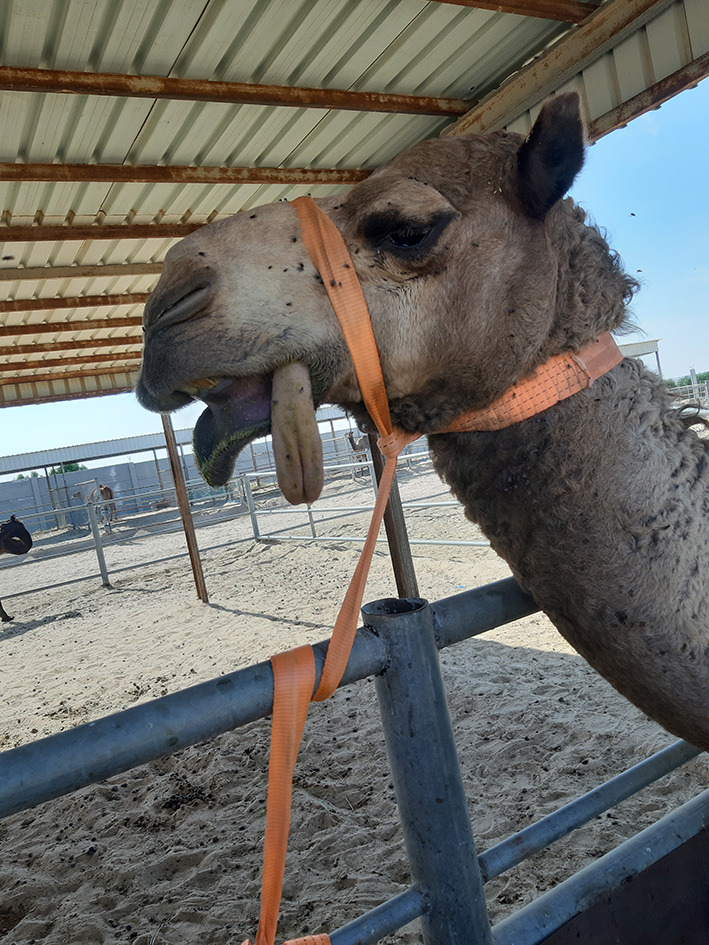
A camel showing moderate degree of sedation (score = 2) post-epidural injection of romifidine (RO) (50 μg/kg).

Camels in the RO and ROLD groups had significantly different sedation scores than those in the LD group (*P* < 0.05, Table 3). LD elicited no sedative effect. Both RO and ROLD induced mild to deep sedation (scores = 1–3), which was noted 5 min after epidural administration (Table 3). The duration of the sedative effect lasted ~210 min post administration of both RO and ROLD. The onset of sedation did not differ between the two treatments (Table 2). However, the RO duration of sedation was significantly longer than ROLD (*P* < 0.001). The maximum sedation detected (score = 3) was recorded between 30 and 90 min after epidural injection of both RO and ROLD. All the animals were quiet and looked to be unconcerned about their surroundings. Dropping of the head and lips, ptosis of eyelids, deviation of the neck and pronounced ears tips separation, drooling of saliva (sialorrhea or hypersalivation), and exposure of the tongue from the mouth were observed (Figure 2). All treated camels were no longer sedated and eating, drinking, and defecating normally 12 h after the epidural injection.

**Table 3:** Sedation scores (median and range) pre-epidural and post-epidural injection of romifidine (RO) (50 μg kg^−1^), romifidine-lidocaine (ROLD) (50 μg and 0.3 mg kg^−1^) and lidocaine (LD) (0.3 mg kg^−1^) in camels (*n* = 18).

**Treatment**	**Time post-administration (minutes)**												
	**0**	**5**	**15**	**30**	**45**	**60**	**90**	**120**	**150**	**180**	**210**	**360**	**720**
RO	0 (0)	1 (1–2)^a^	2 (2–3)^a^	2 (2–3)^a^	2.5 (2–3)^a^	3 (2–3)^a^	2.5 (2–3)^a^	1.5 (1–2)^a^	1 (1–2)^a^	1 (0–1)^a^	0 (0–1)	0 (0–0)	0 (0–0)
ROLD	0 (0)	1 (1–1)^a^	2 (2–3)^a^	2 (2–3)^a^	2 (2–3)^a^	2.5 (2–3)^a^	2.5 (2–3)^a^	2 (1–2)^a^	1 (1–2)^a^	1 (0–1)^a^	0 (0–1)	0 (0–0)	0 (0–0)
LD	0 (0)	0 (0–0)^b^	0 (0–0)^b^	0 (0–0) ^b^	0 (0–0)^b^	0 (0–0)^b^	2 (0–2)^b^	0 (0–0)^b^	0 (0–0)^b^	0 (0–0)^b^	0 (0–0)	0 (0–0)	0 (0–0)
*P* value	1	<0.001	<0.01	<0.001	<0.01	<0.01	<0.05	<0.01	<0.01	<0.03	0.6	1	1

a, b * Variables with different superscript letters at the same column are significantly differ at P < 0.05*.

Mild (score = 1) to moderate (score = 2) ataxia was noted in all treated animals 5 min post-administration and lasted for 180 min (Table 4). However, sever ataxia (score = 3) was noticed in some animals after 60 min and lasted for 90 min. The camels in the ROLD group were more ataxic than those treated with RO and LD (at 30 and 45 min post-administration). Sternal recumbency (Cush position) occurred in three camels 60–90 min after RO (one camel) or ROLD (two camels) administration. Recumbency persisted for 20–30 min, and the camels subsequently stood without support.

**Table 4:** Ataxia scores (median and range) pre-epidural and post-epidural injection of romifidine (RO) (50 μg kg^−1^), romifidine-lidocaine (ROLD) (50 μg and 0.3 mg kg^−1^) and lidocaine (LD) (0.3 mg kg^−1^) in camels (*n* = 18).

**Treatment**	**Time post-administration (minutes)**												
	**0**	**5**	**15**	**30**	**45**	**60**	**90**	**120**	**150**	**180**	**210**	**360**	**720**
RO	0 (0)	0 (0–0)	1 (1–2)	1.5 (1–2)	2 (1–2)	2 (2–3)	2 (2–3)^a^	1 (0–1)	1 (0–1)^a^	0 (0–1)	0 (0–0)	0 (0–0)	0 (0–0)
ROLD	0 (0)	1 (0–2)	1 (1–2)	2 (1–2)	2 (2–2)	2(2–3)	2 (2–3)^a^	1(0–1)	1 (0–1)^a^	0 (0–1)	0 (0–0)	0 (0–0)	0 (0–0)
LD	0 (0)	1 (0–1)	1 (0–2)	1.5 (0–2)	2 (0–2)	2 (0–2)	1 (0–2)^b^	0 (0–1)	0 (0–0)^b^	0 (0–0)	0 (0–0)	0 (0–0)	0 (0–0)
*P* value	1.00	<0.01	0.85	0.73	0.30	<0.08	<0.01	<0.06	<0.05	0.32	1.00	1.00	1.00

a, b *Variables with different superscript letters at the same column are significantly differ at P < 0.05*.

Epidural administration of RO and ROLD treatments resulted in a significant reduction in ruminal contraction in comparison with LD treated group (Table 5). The lowest contraction rate was noticed at 30–150 min post-administration.

**Table 5:** Ruminal contractions (contraction/5 min; mean ± SE) pre-epidural and post-epidural injection of romifidine (RO) (50 μg kg^−1^), romifidine-lidocaine (ROLD) (50 μg and 0.3 mg kg^−1^) and lidocaine (LD) (0.3 mg kg^−1^) in camels (*n* = 18).

**Treatment**	**Time post-administration (minutes)**												
	**0**	**5**	**15**	**30**	**45**	**60**	**90**	**120**	**150**	**180**	**210**	**360**	**720**
RO	2.5 ± 0.22	2.17 ± 0.17^b^	1.67 ± 0.21^ab^	1.00 ± 0.00^b^	1.00 ± 0.00^b^	0.33 ± 0.21^b^	0.83 ± 0.18^b^	0.83 ± 0.17^b^	1.00 ± 0.00^b^	1.33 ± 0.21^b^	1.5 ± 0.22^b^	2.5 ± 0.22^ab^	3.00 ± 0.00
ROLD	2.67 ± 0.21	2.00 ± 0.00^b^	1.5 ± 0.22^b^	0.83 ± 0.21^b^	0.67 ± 0.21^b^	0.17 ± 0.22^b^	1.00 ± 0.00^b^	0.83 ± 0.17^b^	1.00 ± 0.00^b^	1.00 ± 0.00^b^	1.17 ± 0.17^b^	2.17 ± 0.17^b^	2.83 ± 0.17
LD	2.83 ± 0.17	2.83 ± 0.18^a^	2.17 ± 0.17^a^	2.5 ± 0.22^a^	2.67 ± 0.21^a^	2.83 ± 00^a^	2.5 ± 0.34^a^	2.67 ± 0.21^a^	2.33 ± 0.33^a^	2.67 ± 0.21^a^	2.67 ± 0.21^a^	2.83 ± 0.17^a^	2.83 ± 0.16

*Wilks' Lambda test for treatment × time interaction, P < 0.05*.

*Wilks' Lambda test for within treatment (time), P < 0.05*.

*^a, b^ Means with different superscript letters at the same column are significantly differ at P < 0.05*.

**Figure 3:**
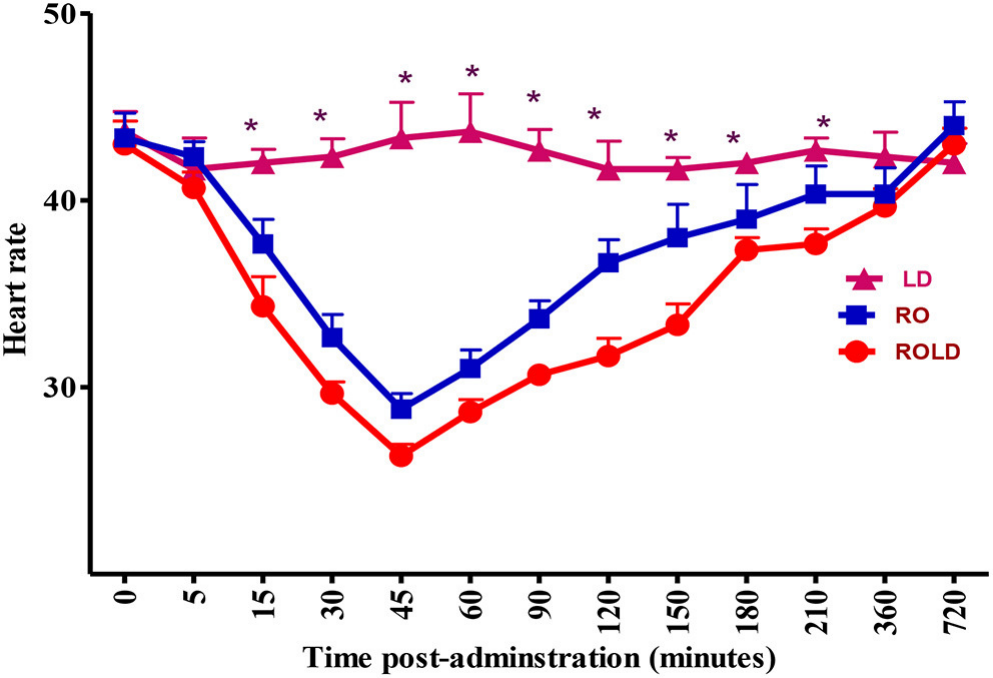
Heart rate (beat/min; mean ± SE) pre-epidural and post-epidural injection of romifidine (RO) (50 μg/kg), romifidine-lidocaine (ROLD) (50 μg and 0.3 mg/kg) and lidocaine (LD) (0.3 mg/kg) in camels (*n* = 18). *Significant at *P* < 0.05.

**Figure 4:**
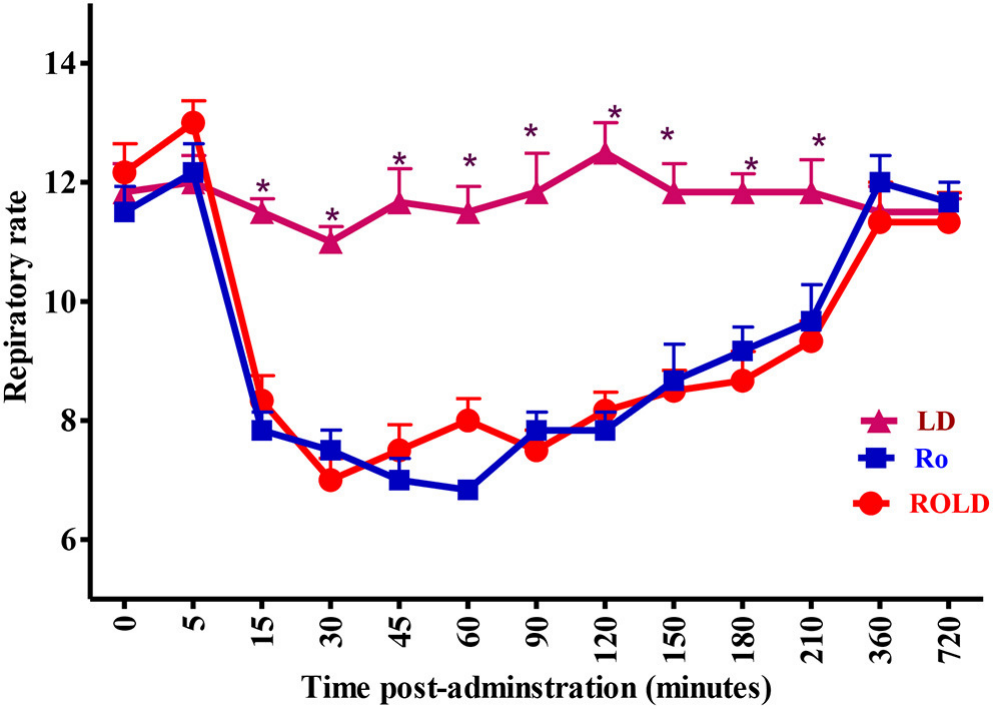
Respiratory rate (respiratory cycle/min; mean ± SE) pre-epidural and post-epidural injection of romifidine (RO) (50 μg/kg), romifidine-lidocaine (ROLD) (50 μg and 0.3 mg /kg) and lidocaine (LD) (0.3 mg/kg) in camels (*n* = 18). *Significant at *P* < 0.05.

Epidural injection of RO and ROLD treatments resulted in a significant reduction in RR and HR (*P* < 0.05) compared to the baseline (Figures 3, 4). In contrast, camels who had received LD did not show any changes in these variables. The lowest HR occurred (*P* < 0.001) at 45–60 min post-injection. HR and RR did not change significantly between RO and ROLD treatments (*P* > 0.05) or during observation time within the same treatment. The RT remained constant compared to the baseline value at all times, and no significant differences were found between the two treatments. The number of ruminal contractions in 5 min significantly decreased at 30–120 min post-administration (*P* < 0.001). At 60 min after administration, the lowest contraction rate was 0.33 ± 0.21 and 0.17 ± 0.22 in 5 min for RO and ROLD treatments, respectively.

Anal sphincter relaxation, ballooning of the caudal part of the rectum was noticed in all camels at 5 and remained until 60, 120, and 150 min post-administration of LD, RO, and ROLD, respectively.

All treated camels showed no clinical evidence of discomfort. Moreover, almost no side effects such as penile prolapse and marked tympany were reported post administration of all treatments. However, in camels receiving RO and ROLD treatments, frequent urination commenced between 90 and 180 min and all camels urinated more than once (range 4–6 times). Also, mild sialorrhea and lacrimation were observed. Moreover, some camels spat foul-smelling regurgitated food at the experimenters at the beginning of the experiment.

## Discussion

In this randomized prospective study, we present the first investigation evaluating the sedative and anti-nociceptive effects of epidural RO, ROLD and LD in camels. In routine practice, camels are poor subjects for general anesthesia due to the difficulty in intubating this species for anatomical oropharyngeal reasons and the increased risk of complications associated with recumbency such as regurgitation, aspiration pneumonia, tympany, and muscular and nerve damage (22). Therefore, most surgical interventions in the camel can be performed under the influence of sedation and locoregional anesthesia. Caudal epidural analgesia is an easy, simple, and cost-effective regional analgesic technique used to perform many surgical procedures such as urethrostomy, rectal prolapse, perineal lacerations, rectovaginal fistula, uterine prolapse, tail amputation, persistent hymen, and transverse vaginal septum (23). By providing anti-nociceptive and sedation scores, the results of this study can assist camel surgeons in assessing the possible clinical application of epidural RO alone or co-administrated with LD for the completion of many surgical and obstetrical interventions, either in the standing or recumbent position.

The total volume and dosage of RO used in this study were determined primarily from previous research conducted in food animals and unpublished pilot studies (2, 18, 20). In addition, all these previous treatments were injected directly into the sacrococcygeal space. However, the epidural space is shallow and easy to enter compared to the first intercoccygeal space. Among large domesticated animals, the camel has the longest average spinal cord, 213.16 cm. It extends from the level of the foramen magnum to the second and third sacral vertebra (24). The cranial extent of spreading and anti-nociception can be enhanced by increasing the volume of the drug administered in the epidural space. As a result, a volume of 20 ml per camel was selected to adjust to a standard volume for use by practitioners.

Very few studies have been published regarding the use of alpha-2 adrenoceptor agonists for epidural analgesia in dromedary camels. Epidural xylazine in camels and South American camelids such as llamas and alpacas have been shown to provide effective anti-nociceptive effects in the tail, anus and perineum with no or minimal adverse effects (12, 13, 15). Epidural administration of alpha-2 adrenoceptor agents resulted in analgesia due to stimulation of both presynaptic and postsynaptic alpha-2 adrenergic receptors in the spinal dorsal horn. This stimulation causes suppression of the central transmission of the afferent nociceptive impulses and a decrease in interneuron transmission of norepinephrine and substance-p, which is involved in nociception, resulting in reduced neural activities and anti-nociception (25, 26). The magnitude of anti-nociception provoked by epidural administration of RO in our study could also be attributed to systemic action following absorption through vascular or lymphatic structures in the epidural space. In camels, intravenous RO has been shown to produce comparable levels of anti-nociception (17). We diluted RO in a relatively large volume of normal saline in our study, allowing the drug to migrate cranially to the sacral, lumbar, and thoracic spinal cord segments, implying that its actions were both local and systemic.

Although pain management is an important part of veterinary medicine, it is usually ignored and underused in camel practice. In addition, camels have an extraordinary ability to bear pain without showing any signs of distress, making recognizing pain scores in camels difficult. The grimace score is a method of evaluating the incidence or intensity of pain in animals based on objective and blinded facial expression scoring, which is also challenging to apply in camels. As a result, the development of tools for assessing pain in camels is required and necessitates further research. In analgesiometry, various pain assessment models for animals have been mentioned (27). Electrical stimulus testing has been used reliably to facilitate an objective assessment of cutaneous nociception in cattle (1, 20); however, in the current study, mechanical stimulation by administering pinpricks and pinching with artery forceps was used to assess the analgesic effects as these are common methods used in animal research (9, 28), especially for aggressive and difficult to control animals such as camel.

Our results suggest that epidural injection of RO and the ROLD combination provides complete anti-nociception of the tail, perineum, inguinal area, caudal aspect of the upper hind limb, flank, chest, and the footpad. These results are similar to those previously reported in ruminants (2, 20, 29). Similarly, previous research in camels reported a significant degree of anti-nociception in the perineum after epidural injection of xylazine, but analgesia was only assessed in the perineal region (15).

In the current study, the onset of anti-nociception after epidural RO administration was rapid, 6.67 ± 0.33 min. This effect was significantly shorter than that observed after epidural injection of xylazine in camels (20.5 ± 3.32). The anti-nociception duration reported in this study after epidural RO and ROLD administration was also longer than LD administration alone, consistent with previously observed results of another alpha-2 adrenoceptor agonist (xylazine) in camels (15). The reduced duration of anti-nociception produced by epidurally injected LD may be attributed to vasodilation and greater absorption of the medication from the spinal cord into the systemic blood circulation caused by LD's sympathetic blockade (30). Furthermore, previous research has shown that xylazine, enhances the anti-nociceptive effects of LD after epidural administration in camels (15, 31).

In both RO and ROLD groups, anti-nociception was first observed in the tail and perineum and then progressed cranially to the flank and distally to the footpad. Furthermore, the tail, perineum, inguinal, caudal aspect of the upper hind limbs, flank, and chest had greater anti-nociception depth and duration than the dorsal metatarsal regions, footpad, and ventral abdominal wall. This observation is most likely related to the cranial migration of a large amount of the drug to the sacral, lumbar and thoracic spinal cord segments. The reason for the duration of anti-nociception of both RO and ROLD treatments on the dorsal metatarsal region and footpad compared to other areas is unknown. The extreme sensitivity of the dorsal metatarsal region and footpad may justify the anti-nociceptive effect's short duration.

In clinical camel practice, alpha-2 agonists are often used to cause sedation and are classified as sedatives and analgesics (17). Sedation and other clinical effects after epidural administration of alpha-2 adrenoceptor agonists are expected to be produced *via* the rapid systemic uptake from the vascular or lymphatic structures in the epidural space and/or dissemination into the cerebrospinal fluid. Cranial spreading to the central nervous system then occurs, producing CNS depression by initiating both the central and peripheral presynaptic and postsynaptic alpha-2 adrenoceptors, blocking the further release of noradrenalin required for awakening (32, 33). Sedation can also be attributed to the inhibition of motivating activity in the locus coeruleus neurons, a small nucleus deep in the pons of the brainstem that is involved in many vital behavioral processes like the sleep-wake cycle and physiological responses to anxiety and stress (34). The high lipid solubility of RO may explain the rapid onset of its sedative effect (35).

The duration of sedation was significantly prolonged with RO (160 ± 6.33 min) than ROLD (141.67 ± 5.43 min). Correspondingly, these findings contrasted with those of a previous investigation in which epidural administration of RO 50 μg/kg in cattle and buffaloes caused similar sedation, but the average length of the effect was longer (extended for at least 720 min) than that found in the current study, this variation may be attributed to the species difference and slow metabolism and the high plasma concentration of RO in cattle and buffaloes after epidural injection in comparison with camels.

We detected a significant severe to slight degree of ataxia in the camels, which could be attributed to the coupled systemic effects of muscular relaxation and sedation of alpha-2 agonists (36). Alpha-2 adrenoceptor agonists particularly inhibit sensory nerve fibers while not affecting motor fibers (37). These results are consistent with those reported after epidural injection of the same RO dose in related species (2, 20, 29, 38) except llamas, in which ataxia did not develop after administration of xylazine and a combination of LD and xylazine (12). From the clinical point of view, in contrast to large ruminants, most surgical interventions in camels have been performed in the sternal recumbency position, so concerns regarding severe ataxia reported in some cases are considered of minor importance.

We detected no significant effect on RT of treated animals after administering all treatments. This observation was similar in the systemic use of RO (17), detomidine (39) and xylazine (36) in camels. Alpha-2 agonists may help maintain body temperature by causing superficial vascular constriction and central recirculation of blood, resulting in less cutaneous heat loss (40). However, a significant decrease in the RT was reported after epidural administration of xylazine in dromedary camels (15), attributed to alpha-2 agonist's generalized sedation, muscle relaxation, central nervous system depression of thermoregulatory centers, and a decrease in basal metabolic rate.

A significant decrease in HR and RR was observed post-administration of both RO and ROLD treatments. The systemic uptake of these drugs, which causes a centrally mediated depression of the respiratory center, could explain the observed decrease in RR (41). Similar findings have been reported in camels post epidural administration of xylazine (15), and in horses and goats post epidural administration of RO (19, 38). Conversely, no significant changes in RR has been observed in large ruminants (2, 20). Bradycardia after epidural administration of RO might be attributed to central stimulation mediated through the vagus nerve and reduction in sympathetic tone due to the decreased presynaptic release of norepinephrine (42). Similar findings have been reported in camels after administration of xylazine (15), and in ruminants and non-ruminants after administration of RO (2, 19, 20, 38).

Ruminal stasis (atony) of camels receiving both RO and ROLD treatments was also observed. A similar finding has been reported in ruminants (2, 20, 38). The risk of ruminal atony (stasis) and subsequent ruminal bloat is considered a significant barrier to using alpha-2 adrenoceptors in ruminants. However, all camels in the current study were eating, drinking, and defecating well, and no camels showed evidence of marked tympany within the 12-h study period.

Frequent micturition was noticed in all cases following RO and ROLD administration. Similar results were observed in ruminants and horses (2, 19, 20, 38). The higher micturition frequencies might be due to the inhibition of antidiuretic hormone release and hyperglycemia due to inhibition of insulin secretion from pancreatic beta cells (43). We also observed mild sialorrhea, similar to that previously recorded in goats and buffalo (2, 38). In addition, cattle given RO had noticeable increases in salivation (20).

The current study has four main limitations. First, we included only clinically normal camels in an experimental model, which may not respond similarly to camels who would require surgery or experience painful conditions. The second limitation is the use of only one dose of RO, which prevents the evaluation of RO's dose-dependent anti-nociceptive and sedative effects in camels. The third one is the lack of negative control group. Finally, a scarcity of knowledge on the pharmacokinetics of RO in camels may make it difficult to identify and explain some of the drugs' clinical effects. Our study's limitations should be considered in future research to reach more definitive conclusions.

## Conclusion

From our results, we concluded that epidural injection of a single dose of RO or the combination ROLD produced a rapid onset and a long duration of complete bilateral caudal epidural analgesia with no adverse effects compared to LD alone. Overall, the epidural RO and ROLD sedation and ataxia scores were acceptable. An epidural RO and ROLD would appear to produce a very effective and acceptable anti-nociceptive effect in the perineal and inguinal regions of camels. Moreover, more research is warranted in the future concerning withdrawal times, adverse effects and to determine whether the analgesia is adequate for specific surgical techniques or alleviating postoperative pain before final recommendations can be made.

## Data Availability Statement

The raw data supporting the conclusions of this article will be made available by the authors, without undue reservation.

## Ethics Statement

The animal study was reviewed and approved by Saudi Arabian Ethical Codes for Studies on Experimental Animals (Approval No. KFU-REC-EA000559).

## Author Contributions

MM designed and performed research, collected and analyzed data, and wrote the paper. AA performed research and collected and analyzed data. SA-R and SE-k analyzed data and wrote the paper. TS performed research and analyzed the data. MK designed and performed research and collected and analyzed data. All authors revised the paper and approved submission.

## Conflict of Interest

The authors declare that the research was conducted in the absence of any commercial or financial relationships that could be construed as a potential conflict of interest.

## Publisher's Note

All claims expressed in this article are solely those of the authors and do not necessarily represent those of their affiliated organizations, or those of the publisher, the editors and the reviewers. Any product that may be evaluated in this article, or claim that may be made by its manufacturer, is not guaranteed or endorsed by the publisher.
